# Outcomes of Migraine and Tension-Type Headache in Children and Adolescents

**DOI:** 10.3390/life11070684

**Published:** 2021-07-13

**Authors:** Jacob Genizi, Ayellet Hendler-Sade, Idan Segal, Ellen Bamberger, Isaac Srugo, Nogah C. Kerem

**Affiliations:** 1 Pediatric Neurology Unit, Bnai Zion Medical Center, Haifa 31048, Israel; dr.idansegal@gmail.COM; 2 Pediatric Department, Bnai Zion Medical Center, Haifa 31048, Israel; ayelleth@gmail.com (A.H.-S.); esbamberger@gmail.com (E.B.); srugoi@gmail.com (I.S.); nogah.kerem@b-zion.org.il (N.C.K.); 3 Bruce Rappaport Faulty of Medicine, Technion, Haifa 31048, Israel; 4 Adolescent Medicine Unit, Bnai Zion Medical Center, Haifa 31048, Israel

**Keywords:** primary headaches, pediatric, outcome, diagnosis, change

## Abstract

The aim of our study was to evaluate the long-term outcomes of pediatric migraine and TTH in a clinical setting. We conducted a cohort study. Pediatric patients who visited the pediatric neurology clinic due to diagnoses of migraine or TTH were contacted by phone 8–10 years after their initial diagnosis and interviewed about their outcomes. Of 147 children, we were able to reach 120 (81%) patients. Of these 120 patients, 59 were seen initially due to migraine and 61 due to TTH. For the migraine patients, headaches improved in 48 (81.4%) and worsened in four (6.8%). Regarding diagnosis at follow-up, 59% still had migraine, 17% had TTH, and 23% were headache-free. Aura and photophobia were significantly associated with persistence of a migraine diagnosis. For the TTH patients, headaches improved in 49 (81.7%) and worsened in nine (15.0%). Regarding diagnosis at follow-up, 36.7% still had TTH, 18.3% had migraine, and 45% were headache-free. Of the patients with TTH, 36.7% retained their initial diagnosis compared to 59.3% among the migraine patients. Most pediatric patients presenting with migraine or TTH will experience a favorable outcome over 10 years, with TTH patients having twice the chance of complete resolution.

## 1. Introduction

Headaches are very common in children and adolescents. Indeed, they are the most common pain complaint among children and adolescents seeking medical advice [1]. Migraine and tension-type headache (TTH) are the most common headache syndromes in children [2]. Although the prevalence of migraine during the preschool years is as low as 3%, it increases by the early school years to 11%, and during the high school years, it is as high as 23% [3]. The overall mean prevalence of migraine was recently estimated to be between 7.7% and 9.1% [2,3,4]. Among prepubertal children, boys have more episodes of migraine than girls, but after puberty migraine headaches occur more frequently in girls, as is seen among adults [2]. The mean age of onset for migraine is 7 years for boys and 11 years for girls [1]. Approximately a third of children and adolescents who have migraines report experiencing aura before onset of the headache [4,5,6]. Most have visual symptoms that begin gradually and last for several minutes (typical aura). The most frequent of these are binocular visual impairment with scotoma (77%), distortion or hallucinations (16%), and monocular visual impairment or scotoma (7%) [5]. The prevalence of TTH is not much different from migraine, varying from 9 to 18% [5] and increasing with age.

Headache in children and adolescents can cause impaired psychosocial functioning in different areas of life, including family, activities, and school tasks [7]. Adolescents with headache are at heightened risk of developing additional physical problems in adulthood, as well as mental difficulties such as anxiety and depression [7,8].

With respect to the long-term prognosis, Forsythe and Hockaday [9] reported that about one-third of pediatric patients with migraine experience long-term remission, while Hockaday [10] reported remission in 25% of childhood migraine patients and improvement in another 48%. Congdon and Forsythe [11] emphasized that although 34% showed remission after 10 years, remission did not occur in any patients after 18 years of age. Sillampää [12] differentiated between onset of migraine before and after eight years of age, with a better prognosis for the former, especially among boys. Long-term prognosis of TTH in the pediatric age group has not yet been studied thoroughly.

The purpose of the present study was to evaluate outcomes 10 years after the first clinic visit for children and adolescents with migraine vs. TTH and to identify risk factors for the future course of these common childhood headache syndromes. We hypothesized that the prognosis for both types of pediatric headaches, migraine and TTH, would be good, with no major differences between them.

## 2. Patients and Methods

This was a cohort study. Children and adolescents who visited the pediatric neurology clinic at Bnai Zion Hospital due to headache during the years 2007–2008 were contacted by phone, on average 10 years after their initial clinic visit. Written informed consent for patient information to be published was provided by the patients and/or a legally authorized representative. The study was approved by the Bnai Zion IRB (BNZ 109-18).

Data were collected during the phone interviews via a structured questionnaire and included demographics; patients’ and families’ medical history; and headache history, past and current (e.g., age at onset, location, quality, frequency, duration of episodes, aura, associated symptoms, and treatment). Migraine, TTH, and other headache types were diagnosed according to the ICHD-3 criteria [13] (the initial diagnosis was reassessed accordingly).

Statistical analyses were performed using chi-square tests or Fisher’s exact test where appropriate for the categorical data, and using independent t-tests for the continuous variables. Odds ratios and 95% confidence intervals were calculated. Statistical significance was considered to be *p* < 0.05. Multiple logistic regression analysis was performed using gender, age, and headache diagnosis. Following this, a stepwise logistic regression was conducted to evaluate two-way interactions as potential predictors. Statistical analysis was performed using SPSS software version 21 (SPSS, Chicago, IL, USA).

## 3. Results

**Figure 1:**
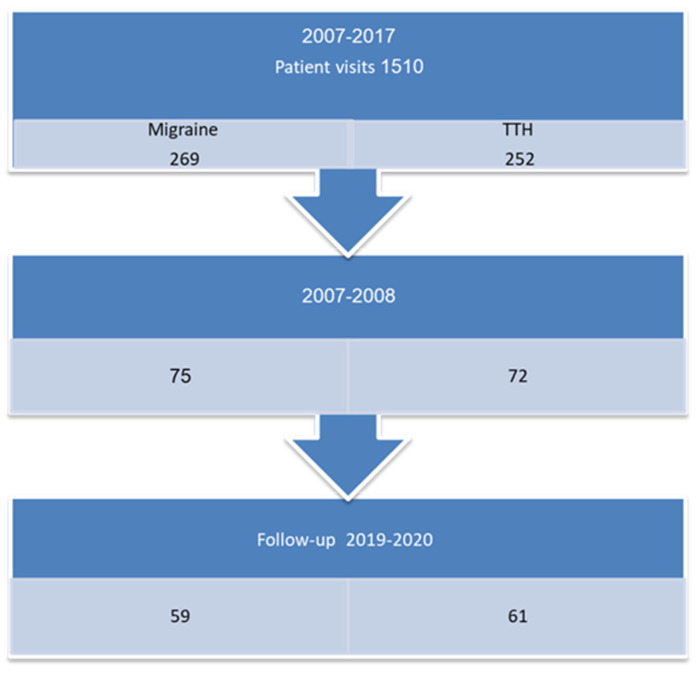
Study flow chart.

Of 147 children seen at our pediatric headache clinic in 2007–2008 due to headache, we were able to reach 120 (81%) patients for follow-up (Figure 1).

Fifty-nine patients fulfilled the criteria for migraine during their first clinic visit (see Table 1 and Table 2 for these patients’ present demographics). Of these, 35 were males, and mean age at presentation was 12 years of age. Mean time elapsed since the initial diagnosis was 9.3 years (range 8–10 years). Fifty-six patients were initially diagnosed with episodic migraine. At follow-up, 48 (81.4%) patients experienced less frequent headache episodes, four (6.8%) had more frequent episodes, and for seven (11.9%), the frequency of the headache episodes remained stable.

For migraine patients, we found no gender differences in either age at onset or age of improvement. Patients without a family history of migraine tended to suffer migraines for significantly fewer years compared to those with a positive first-degree family history of migraine (*p* < 0.06). However, a positive family history of migraine was not associated with age at onset or with age of improvement.

The diagnosis of migraine remained stable over the years for 35 (59.3%) of the patients in the migraine group and changed to TTH in 10 patients (16.9%). Another 14 patients (23%) were headache-free at the time of follow-up. Table 3 presents clinical data for these patients.

A significantly higher percentage of patients who were still diagnosed as having migraine at follow-up had initially presented with aura (χ2 = 4.02, *p* < 0.05) or photophobia (χ2 = 8.41, *p* < 0.02), compared to those who were currently diagnosed with TTH (χ2 = 8.19, *p* < 0.004). Other variables such as gender, family history, age at onset of migraine episodes, and number of years with migraine episodes were not associated with the current diagnosis. No significant difference in headache frequency was found between patients currently diagnosed with migraine and those currently diagnosed with TTH (χ2 = 4.33, *p* > 0.11).

Sixty-one patients fulfilled the criteria for TTH during their first clinic visit. Of these, 20 were males (32%), mean age at presentation was 12 years of age, and mean time between initial clinic visit and follow-up was 9.2 years (range 8–10 years). See Table 3 for demographic and clinical characteristics of the TTH patients.

Fifty-four patients had episodic TTH. Forty-nine (81.7%) of the patients in this group experienced less frequent episodes of headache over the years, while for nine (15.0%), the frequency of episodes increased, and for two (3.3%), the frequency of headache episodes did not change. Gender was not found to be a significant contributor to the change in frequency of headache episodes in the TTH group.

The diagnosis of TTH remained unchanged at the follow-up interview for 22 (36.7%) of the patients in this group, while 11 (18.3%) met the criteria for migraine at follow-up, and the remaining 28 (45%) reported being free of headache episodes. Table 4 and Table 5 present selected demographic and clinical data for these patients.

Comparing the patients who initially presented with migraine to those who initially presented with TTH reveals that there were more males in the migraine group (*p* < 0.004), and age at onset of headache syndrome tended to be younger in the migraine group (*p* < 0.09). Time until resolution of headache episodes was significantly longer for patients in the migraine group (*p* < 0.02), and TTH patients were twice as likely to be headache-free at follow-up compared with migraine patients (45.0% vs. 23.7%; *p* < 0.02). Only 36.7% of the patients with TTH kept the initial diagnosis at follow-up compared to 59.3% among the migraine patients (*p* < 0.01). See Table 5 for a comparison between the migraine and TTH groups.

## 4. Discussion

This study followed-up a group of children and adolescents with TTH and migraine headache 8–10 years following their initial diagnosis. Since pediatric primary headache syndromes are very common and may cause significant morbidity in both physical and psychosocial functioning, including anxiety and depression [7], the long-term outcome is crucial.

Only a few studies have addressed the long-term prognosis of childhood headache syndromes. Moreover, of these, most studies have focused on pediatric migraine [8,11,14,15,16,17,18,19], with very few evaluating TTH as well [7,20,21].

Our findings show that among pediatric-onset migraine patients, 81% improved in regard to the existence and frequency of headache episodes over an average period of 9.3 years, with 24% enjoying complete remission. However, 76% still reported suffering headache episodes, with 60% still meeting migraine criteria and 16% now fulfilling the criteria for TTH.

Our findings are in accordance with those of Camarda et al. [17], who found in a five-year follow-up of 64 pediatric patients with migraine that migraine diagnosis persisted in 56.2%, changed to TTH in 12.5%, and remitted in 19%. Monastero et al. [18], following 55 patients from the same group for 10 years, reported that 42% still had migraine, 20% had shifted to TTH, and 38% had remission of their headache. Monastero et al. also reported that family history predicts the persistence of migraine diagnosis. In our study, in contrast to that of Monastero et al., family history was not associated with persistence of headache diagnosis (*p* = 0.84). We found that migraine with aura and photophobia was significantly associated with persistence of migraine diagnosis.

Bille [14], in a very long-term follow-up study of 40 years, tracked 73 children with migraine. Bille found that 23% of the patients studied were headache-free by the age of 25, which is very much in accord with our findings, and at 16 years of follow-up 40% were headache-free. However, after 40 years of follow-up, only 47% were headache-free, and more than half the patients with pediatric migraine still had migraine at the age of 50. In that study, males had a better prognosis for becoming headache-free than females, in contrast to our findings, where gender did not play a role in the likelihood of remission.

Among the TTH patients, our findings indicate that 82% improved during the average time of 9.7 years until follow-up, with 45% experiencing complete remission of headache episodes. Of the 55% who continued to suffer headache episodes, 37% still fulfilled the criteria for TTH, and 18% had shifted to a diagnosis of migraine.

Guidetti and Galli [7], in an 8-year follow-up, reported improvement in 95% of children with TTH, with 34% experiencing complete remission. Diagnosis of TTH persisted in 26.3% and changed to migraine in 11%. Males had a much better prognosis for becoming headache-free (52.5%) compared to females (21.7%), among both migraine and TTH patients. As previously indicated, our study found no gender differences in headache prognosis. Mazzotta [20], in a large 3-year follow-up study of 442 pediatric patients with migraine and TTH, also reported no gender differences that might predict elimination of headache syndromes. Similar findings were reported by Balottin [21], who followed young children with primary headaches for several years.

In our study, approximately 9 years after first presentation to the pediatric neurology clinic, patients with TTH had a much better prognosis for being headache-free than did patients with migraine (44% vs. 24%, respectively). Mazzotta’s [20] large (442 patients) 3-year follow-up study also found that only 17.4% of patients with migraine were headache-free by the time of follow-up, in comparison to 32.9% of patients with TTH. A difference in recovery between migraine and TTH was also reported by Guidetti and Galli [7], who found that 28.1% of migraine patients and 44.4% of TTH patients had recovered after an 8-year follow-up.

Our study indicates that in 17–18% of our patients, the headache diagnosis changed from migraine to TTH, and vice versa. A few studies support these findings. Guidetti and Galli [7], in their 8-year follow-up, found a shift from migraine to TTH in 26% of their patients, and from TTH to migraine in 11%. Monastero et al. [18], in a 10-year follow-up, found that migraine transformed to TTH in 20%. Battistel et al. [22] reported that in 14% of young children diagnosed with migraine without aura, the migraine had evolved by adolescence to episodic tension-type headache. However, in contrast to our findings, TTH did not evolve into migraine in any of their patients, even without aura.

Several possible explanations suggest themselves for these shifts in the diagnosis of pediatric headache patients over time, from migraine to TTH and vice versa. The first is an incorrect initial diagnosis. Obtaining a complete and accurate headache history from a young child may be challenging, and parents’ reports could influence the doctor’s impression and, therefore, the patient’s diagnosis. However, if this were the case, we should have seen differences in the diagnosis shift with time between younger and older children–something that is not evident in either our study or others reported in the literature. Another possible explanation is that the shift can be attributed to hormonal changes during puberty, much as the prevalence of migraine is similar between prepubertal boys and girls, but higher in females than in males post-puberty. Neurogenic inflammatory molecules, including calcitonin gene-related peptide (CGRP) and substance-P (SP), play a major role in migraine pathogenesis, and they are affected by the release of the female sex hormones, estrogen, and progesterone [23].

Finally, some argue that migraine and episodic TTH in childhood are both part of the same headache syndrome or, more precisely, two ends of the same continuum [8,24,25]. This “continuum theory” of headache is also supported by those children and adolescents who present clinically with “mixed headache syndrome,” meaning a combination of TTH and migraine (mostly without aura). In a previous study [26], we found a clinical overlap of 24% between TTH and migraine.

## 5. Limitations

Our study is based on the experience of one clinic at a single center, with a relatively small number of patients.

## 6. Conclusions

In a ten-year follow-up study of pediatric patients diagnosed with either migraine or TTH, most patients had a favorable outcome, with improvement of their headache episodes. Children diagnosed with TTH had a better prognosis for being headache-free at follow-up than those diagnosed initially with migraine. Gender and family history had no impact on prognosis, but having migraine with aura and/or photophobia during childhood was a predictor for persistence of symptoms 10 years post initial diagnosis of migraine. Our findings also suggest that 16–18% of patients diagnosed with migraine or TTH during childhood will see their diagnosis changed within 10 years.

## 7. Patents

This section is not mandatory but may be added if there are patents resulting from the work reported in this manuscript.

## Figures and Tables

**Table 1:** Comparison between initial migraine group and initial TTH group.

Initial Diagnosis	Migraine N = 59 (%)	TTH N = 61 (%)	*p*
Gender			
Male Female	35 (59.3) 24 (40.7)	20 (32.8) 41 (67.2)	0.004
Age at first visit	12.0 ± 3.4 (12; 5.5–18.0)	11.6 ± 3.11 (12; 4.6–18.0)	0.48
Age started	10.7 ± 3.7 (11; 4–18)	11.8 ± 3.1 (12; 4.5–18)	0.09
Years suffered (mean; range)	4.1 ± 3.3 (3.25; 0–14)	2.6 ± 2.6 (2; 0–9)	0.02
Family history of headache	42 (73.7)	29 (47.5)	0.004
ADHD	24 (41.4)	26 (42.6)	0.89
Change in headache frequency:			
Improved Worsened No change	48 (82.8) 4 (6.9) 6 (10.3)	49 (81.7) 9 (15.0) 2 (3.3)	0.14
Frequency of pain (events per year) (mean; range)	36.8 ± 49.7 (24; 1–90)	58.5 ± 49.7 (25; 1–110)	0.65

**Table 2:** Demographic data of TTH patients.

	All N = 61 (%)	Male N = 20 (%)	Female N = 41 (%)	*p*
Age at first visit (mean; range)	11.6 ± 31 (12; 4.6–18.0)	11.0 ± 3.0 (11.5; 6.2–18)	12.0 ± 3.2 (12; 4.6–18.0)	0.24
Age at follow-up	17.6 ± 4.0 (18; 8–25)	16.3 ± 3.6 (15.5; 9.9–25)	18.2 ± 4.1 (18; 8–25)	0.08
Age started (N = 55)	11.8 ± 3.1 (12; 4.5–18)	10.8 ± 2.9 (11.5; 6–18)	12.3 ± 3.1 (12; 4.5–18)	0.09
Age improved (N = 45)	13.4 ± 3.6 (13; 7–21)	12.6 ± 2.4 (13; 8–17)	13.8 ± 4.0 (14; 7–21)	0.32
Years suffered	2.6 ± 2.6 (2; 0–9)	2.3 ± 2.7 (1; 0–9)	2.7 ± 2.5 (2; 0–9)	0.61

**Table 3:** Migraine patients by current diagnosis.

Current Diagnosis	Migraine N = 35 (%)	TTH N = 10 (%)	No Headache N = 14 (%)	*p*
Age at first visit (mean; range)	12.8 ± 3.4 (14; 5.5–18.0)	11.3 ± 3.7 (11; 5.9–18)	10.8 ± 3.0 (11; 6.5–18.0)	0.14
Age migraine started	11.4 ± 3.5 (12; 5–18)	9.1 ± 4.5 (7; 4–17)	10.0 ± 3.2 (10.25; 5–18)	0.18
Years suffered	3.4 ± 3.1 (2.0; 0–13)	5.7 ± 4.3 (6.0; 0–14)	4.8 ± 2.8 (4.5; 0.5–8.0)	0.16
Gender:				
Male Female	19 (54.3) 16 (46.7)	7 (70.0) 3 (30.0)	9 (64.3) 5 (35.7)	0.61
Family history	26 (76.5)	7 (70.0)	9 (64.3)	0.84
Aura	6 (17)	0 (0.0)	0 (0.0)	0.05
Photophobia	22 (63)	1/9 (11.1)	7 (50.0)	0.02
Frequency of headaches	58.5 ± 39.8 (12; 2–100)	19.0 ± 18.7 (12; 1–50)	0	0.11

**Table 4:** Demographic data of migraine patients.

	All (N = 59)	Male (N = 35)	Female (N = 24)	*p*
Age at first visit (mean; range)	12.0 ± 3.4 (12; 5.5–18.0)	11.3 ± 3.5 (11; 5.5–18.0)	13.1 ± 3.2 (13; 7.1–18.0)	0.05
Age migraine started (N = 58)	10.7 ± 3.7 (11; 4–18)	10.1 ± 3.5 (10; 4–18)	11.5 ± 3.8 (12; 4–18)	0.17
Age improved (N = 42)	14.6 ± 4.2 (15; 7–26)	14.5 ± 4.0 (15; 6–23)	14.8 ± 4.7 (15; 9–26)	0.84
Years suffered	4.1 ± 3.3 (3.25; 0–14)	4.4 ± 3.4 (4.; 0–14)	3.7 ± 3.2 (3; 0–10)	0.47

**Table 5:** TTH patients by current diagnosis.

Current Diagnosis	Migraine N = 11 (%)	TTH N = 22 (%)	No Headache N = 28 (%)	*p*
Age at first visit (mean; range)	11.6 ± 2.8 (12; 6.9–16.0)	12.4 ± 3.6 (12; 5.5–18)	11.0 ± 2.9 (11; 4.6–16.0)	0.34
Age headaches started	11.2 ± 3.0 (11; 6–16)	13.1 ± 3.1 (13; 8–18)	11.1 ± 2.82 (11.25; 4.5–16)	0.06
Years suffered	4.2 ± 2.6 (5.0; 0.5–8.0)	3.1 ± 2.8 (2.0; 0–9)	1.5 ± 1.9 (1; 0.0–8.0)	0.009
Gender:				
Male Female	3 (27.3) 8 (72.7)	8 (36.4) 14 (63.6)	9 (33.3) 18 (66.7)	0.87
Frequency of headaches	42.4 ± 42.4 (35; 4–150)	88.2 ± 125.1 (12; 1–300)	0	0.67

## Data Availability

Data are available at the Bnai Zion Medical Center archives.
